# Phytoestrogens and Breast Cancer Prevention: Possible Mechanisms of Action

**DOI:** 10.1289/ehp.10538

**Published:** 2008-01-16

**Authors:** Sarah M. Mense, Tom K. Hei, Ramesh K. Ganju, Hari K. Bhat

**Affiliations:** 1 Department of Environmental Health Sciences, Mailman School of Public Health, Columbia University, New York, New York, USA; 2 Division of Experimental Medicine, Beth Israel Deaconess Medical Center, Harvard Medical School, Boston, Massachusetts, USA

**Keywords:** breast cancer, catechol estrogen, catechol-*O*-methyltransferase, chemoprevention, cytochrome P450, estrogen, estrogen receptor, phytoestrogen

## Abstract

**Objective:**

Phytoestrogens display an array of pharmacologic properties, and in recent years investigation of their potential as anticancer agents has increased dramatically. In this article we review the published literature related to phytoestrogens and breast cancer as well as suggest the possible mechanisms that may underlie the relationship between phytoestrogens and breast cancer.

**Data sources:**

Electronic searches on phytoestrogens and breast cancer were performed on MEDLINE and EMBASE in June 2007. No date restriction was placed on the electronic search.

**Data extraction:**

We focused on experimental data from published studies that examined the characteristics of phytoestrogens using *in vivo* or *in vitro* models. We also include human intervention studies in this review.

**Data synthesis:**

We evaluated evidence regarding the possible mechanisms of phytoestrogen action. Discussions of these mechanisms were organized into those activities related to the estrogen receptor, cell growth and proliferation, tumor development, signaling pathways, and estrogen-metabolizing enzymes.

**Conclusions:**

We suggest that despite numerous investigations, the mechanisms of phytoestrogen action in breast cancer have yet to be elucidated. It remains uncertain whether these plant compounds are chemoprotective or whether they may produce adverse outcomes related to breast carcinogenesis.

Breast cancer is an important public health problem worldwide. In the United States, breast cancer represents the most common neoplasm and the second most frequent cause of cancer death in women (American Cancer Society 2006). Steroidal estrogens have been implicated in the etiology of breast cancer and have been added to the list of known human carcinogens [International Agency for Research on Cancer (IARC) 1999, 1987; National Toxicology Program (NTP) 2002]. Estrogens are suggested to cause breast cancer by stimulating cell growth and proliferation through receptor-mediated processes and via their genotoxic metabolites (Cavalieri et al. 2006; Yager and Davidson 2006). Phytoestrogens are a class of plant-derived compounds that are structurally similar to mammalian estrogens (Sirtori et al. 2005). Ecologic observations indicate that the incidence of breast cancer is much lower in Asian women, who consume significantly higher amounts of phytoestrogens than Western women (Adlercreutz 2002). Second- and third-generation descendants of women who migrated to Western countries from Asia have breast cancer risks similar to those of women in the host country, suggesting that lifestyle and not genetic factors explain the low breast cancer risk observed in Asian women (Probst-Hensch et al. 2000; Usui 2006). However, despite recent attention related to the putative chemoprotective properties of phytoestrogens, epidemiologic studies have produced inconsistent results, and the relationship between phytoestrogens and breast cancer remains enigmatic (Gikas and Mokbel 2005; Messina et al. 2006; Peeters et al. 2003; Trock et al. 2006). Moreover, the possible mechanisms of phytoestrogen action in breast cancer have yet to be resolved.

## Phytoestrogen Classification

**Figure 1:**
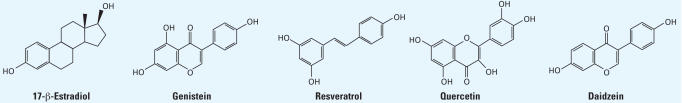
Chemical structures of E_2_ and the phytoestrogens resveratrol, genistein, quercetin, and daidzein.

Phytoestrogens are biologically active phenolic compounds of plant origin that structurally mimic the principal mammalian estrogen 17β-estradiol (E_2_; Figure 1) (Sirtori et al. 2005). Shared structures include a pair of hydroxyl groups and a phenolic ring, which is required for binding to estrogen receptors (ER)-α and ER-β, and the position of these hydroxyl groups appears to be an important factor in determining their abilities to bind the ERs and activate transcription (Le Bail et al. 2000). Four main classes of compounds are currently recognized as phytoestrogens—the isoflavones, stilbenes, coumestans, and lignans (Moon et al. 2006; Sirtori et al. 2005). These types of phytochemicals are some of the most prevalent compounds found in fruits, vegetables, legumes, and tea and are generally concentrated in the fruit skin, bark, and flowers of plants (Moon et al. 2006). Resveratrol, daidzein, quercetin, and genistein represent four of the most commonly ingested and most intensely studied phytoestrogens (Figure 1).

In East and Southeast Asia, the average daily intake of phytoestrogens is estimated to be between 20 and 50 mg (Adlercreutz 1998; Sirtori et al. 2005). In contrast, the typical diet of an adult in the United States contains only 0.15–3 mg phytoestrogens per day, and in Europe the average daily phytoestrogen consumption is estimated to be even lower, falling between 0.49 and 1 mg (Adlercreutz 1998; Sirtori et al. 2005). According to various epidemiologic studies, plasma isoflavone concentrations range from 2 μM (Japanese men) to 5 nM (Finnish study subjects); however, local tissue phytoestrogen concentrations are suggested to be 2–3 times higher than plasma levels (Adlercreutz et al. 1993; Arai et al. 2000; Morton et al. 2002; Uehar et al. 2000).

## Phytoestrogens and Breast Cancer Risk

The importance of estrogens in the etiology of breast cancer is widely recognized (Bhat et al. 2003; Cavalieri et al. 2006; Yager and Davidson 2006). Estrogens have been implicated in the initiation and promotion stages of breast cancer, and lifetime estrogen exposure is a major risk factor for breast cancer development (Yager and Davidson 2006). Estrogens exert their carcinogenic effects via ER-dependent mechanisms as well as their genotoxic metabolites (Bhat et al. 2003; Cavalieri et al. 2006; Yager and Davidson 2006).

Epidemiologic evidence suggests that diet and nutrition can influence cancer development, and women living in Asia, where diets have traditionally included soybean products, report fewer postmenopausal symptoms and experience fewer breast cancers than women in Western countries (Adlercreutz 2002; Nichenametla et al. 2006; Usui 2006). More specifically, Asian women have a 3-fold lower breast cancer risk than women in the United States, independent of body weight (Ursin et al. 1994). Furthermore, serum concentrations of E_2_ are 40% lower in Asian women compared with their Caucasian counterparts (Peeters et al. 2003). Thus, environmental and dietary factors may explain at least some of the discrepancy in breast cancer risk between populations (Adlercreutz 2002; Nichenametla et al. 2006). The assertion that dietary and lifestyle factors may be partially responsible for the low breast cancer risks detected in Asian women is supported by observations in Asian women who immigrate to Western countries. The second- and third-generation descendants of women who migrated from Asia to Western countries have breast cancer risks similar to those of women in the host country, suggesting that lifestyle and not genetic factors explain the low breast cancer risk of women in Asia (Probst-Hensch et al. 2000; Usui 2006).

Phytoestrogens exhibit a wide array of pharmacologic properties, and recently, interest in the potential benefits of diets high in phytoestrogens has intensified, especially those related to chemoprevention. The link between phytoestrogens and breast cancer prevention has been the subject of numerous studies, and the epidemiology of breast cancer in relation to phytoestrogen consumption has recently been extensively reviewed (Adlercreutz 2002; Messina et al. 2006; Ziegler 2004). Generally, epidemiologic studies have been inconclusive, and the relationship between phytoestrogens and breast cancer prevention remains uncertain (Messina et al. 2006; Trock et al. 2006; Ziegler 2004). Some studies have revealed the modest protective effects of phytoestrogens; others have detected no association between phytoestrogen intake and breast cancer risk; and a few have reported marked protective effects (Hirohata et al. 1985; Hirose et al. 1995; Key et al. 1999; Nomura et al. 1978, 1985; Trock et al. 2006; Wu et al. 1996; Yuan et al. 1995). A recent review of 21 case–control and 15 prospective studies concluded that there is no clear evidence that phytoestrogen intake influences the risk of developing breast cancer (Gikas and Mokbel 2005). Nevertheless, some evidence suggests that soy intake must be high during certain windows of development, specifically prepubescence, in order to gain the protective effect of phytoestrogens (Hirayama 1990a, 1990b; Key et al. 1999; Lamartiniere 2000; Shu et al. 2001; Wakai et al. 1999; Wu et al. 1996). Despite intense investigation, it remains unclear whether phytoestrogens are actually chemoprotective agents or whether their presence is simply a biomarker indicative of a healthy diet.

## Phytoestrogens and Estrogen Biosynthesis

Various dietary intervention studies in humans have examined the effects of phytoestrogens on estrogen biosynthesis and estrogen biosynthetic enzymes. Neither a 2-year study in which 220 premenopausal women consumed 100 mg isoflavones per day nor a short-term study in which premenopausal women consumed 38 mg isoflavones per day revealed significant alterations in steroid hormone levels or menstrual cycle length (Hargreaves et al. 1999; Maskarinec et al. 2002). In contrast, other researchers have reported decreased plasma concentrations of follicle-stimulating hormone (FSH), luteinizing hormone (LH), E_2_, and progesterone as well as decreased serum concentrations of E_2_ and estrone following increased phytoestrogen consumption (Duncan et al. 1999; Kumar et al. 2002; Lu et al. 2000a; Nagata et al. 1998). Another study, which evaluated pre-menopausal women after consumption of isoflavone-supplemented diets for three menstrual cycles, reported that isoflavone intake decreased urinary excretion of E_2_, estrone, estriol, and total estrogens (Xu et al. 1998). Moreover, the isoflavone diet increased the ratio of 2-hydroxyestrone to 16α-hydroxyestrone and decreased in the ratio of genotoxic estrogens to total estrogens (Xu et al. 1998). A separate study identified a 27% increase in the ratio of 2-hydroxyestrone to 16α-hydroxyestrone in women given an isoflavone-rich diet compared with women on an isoflavone-free diet (Lu et al. 2000b). Furthermore, in women consuming 40 mg isoflavones each day for 3 months, the average menstrual cycle length was increased 3.52 days, and the follicular phase of the cycle was increased 1.46 days on average (Kumar et al. 2002). The implication of increased menstrual cycle length is a decrease in the total lifetime number of cycles, thereby minimizing the exposure of breast epithelial cells to estrogens.

The decrease in circulating estrogen concentrations after phytoestrogen consumption may be a result of interference with estrogen biosynthetic enzymes, namely cytochrome P450 19 aromatase (Cyp19) and 17β-hydroxysteroid dehydrogenase (HSD) (Rice and Whitehead 2006). Cyp19 catalyzes the conversion of androstenedione and testosterone to estrone (E_1_) and E_2_, respectively (Thompson and Siiteri 1974). HSD catalyzes the inter-conversion of the relatively inactive 17β-keto steroids, such as estrone and androstenedione, to active 17β-hydroxyl steroids such as E_2_ and testosterone (Gunnarsson et al. 2003). In the breast tissue of postmenopausal women, HSD and Cyp19 are responsible for the local production of estrogens, and overexpression or increased activity of these enzymes is associated with breast cancer (Li et al. 1998; Pasqualini et al. 1996; Yue et al. 2001).

Among the phytoestrogens, flavones, and flavonones are the most potent inhibitors of Cyp19 aromatase, whereas the isoflavones are relatively weaker aromatase inhibitors. Several phytoestrogens, including 7-hydroxyflavone, apigenin, chrysin, and hesperetin were found to be effective aromatase inhibitors in human placental microsomes, with IC_50_ (concentration of phytoestrogens that reduces enzyme activity by 50%) values ranging from 0.3 to 3.0 μM (Jeong et al. 1999; Le Bail et al. 2000). In H295R human adrenocortical carcinoma cells, both flavones and flavonones exerted inhibitory effects on aromatase (Sanderson et al. 2004). Similarly, quercetin, genistein, and daidzein suppressed the transcription of *Cyp19* mRNA in human granulosa luteal cells (Rice et al. 2006). Although isoflavones are generally weak aromatase inhibitors, isoflavone mixtures displayed an increased ability to inhibit aromatase activity and transcription compared with any of the isoflavones alone (Rice et al. 2006). A mixture of genistein, daidzein, and biochanin A almost completely eliminated transcription of *Cyp19* mRNA and significantly reduced Cyp19 enzyme activity (Rice et al. 2006). Resveratrol exerted both competitive and noncompetitive inhibitory effects on aromatase activity in MCF7 cells stably transfected with Cyp19, with an IC_50_ value of approximately 25 μM (Wang et al. 2006). Similarly, resveratrol suppressed transcription of *Cyp19* mRNA in SK-BR-3 breast cancer cells. In contrast, genistein increased aromatase activity in H295R cells and in isolated rat follicles (Myllymaki et al. 2005; Sanderson et al. 2004). In addition to their interactions with Cyp19, phytoestrogens have been shown to inhibit HSD (Le Bail et al. 1998). For example, genistein decreased HSD activity in human placental microsomes, genital skin fibroblasts, granulosa luteal cells, MCF7 breast cancer cells and T47D breast cancer cells (Brooks and Thompson 2005; Evans et al. 1995; Le Bail et al. 2000; Whitehead et al. 2002). In MCF7 cells, genistein inhibited HSD-catalyzed E_2_ production by 59% (Brooks and Thompson 2005).

**Figure 2:**
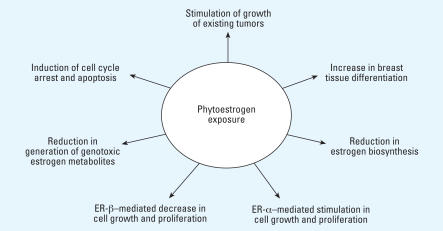
Summary of potential actions of phytoestrogens. Arrows indicate possible functions of phytoestrogens.

Given the carcinogenic properties of endogenous estrogens, reducing their levels in the body by inhibition of steroidogenic enzymes such as Cyp19 and HSD would protect against breast cancer development (Figure 2; Appendix 1). Thus, although studies have not detected consistent changes in hormone levels after phytoestrogen intake and the overall health effects of phytoestrogen exposure remain unclear, these plant compounds may decrease lifetime exposure to estrogens, via two mechanisms, namely by decreasing estrogen biosynthesis and by increasing menstrual cycle length.

## Phytoestrogens and the Estrogen Receptors

The estrogen receptors ER-α and ER-β function as ligand-activated transcription factors that initiate transcription by translocating to the nucleus and binding to estrogen response elements (ERE) in the promoter regions of target genes (McDonnell 2004). The actions of ER-α and ER-β on gene transcription can be opposite, depending on cell context (Koehler et al. 2005). It is thought that ER-βmay impact estrogen action by directly modulating gene transcription or by modulating ER-α activity in tissues that express both ER subtypes (Hall and McDonnell 1999). ER-β can function as a transcriptional inhibitor or activator, depending on the agonist concentration, such that different patterns of gene expression are produced at different agonist concentrations (Hall and McDonnell 1999). Studies in MCF7 cells suggest that ER-β is not necessary for proliferation and that ER-β opposes the proliferative effects exerted by ER-α (Koehler et al. 2005; Omoto et al. 2003; Strom et al. 2004). The interactions between the two main ERs and their specific cofactors provide a mechanistic basis for the tissue-selective actions of estrogens (McDonnell 2004). The ratio of ER-α to ER-β is a prognostic marker in breast tumors, such that ER-β expression is indicative of more benign tumors, whereas ER-α indicates malignant, aggressive tumors (Balfe et al. 2004; Shaaban et al. 2003).

Many phytoestrogens, including resveratrol, genistein, daidzein, and quercetin, have been shown to bind both ER-α and ER-β and to induce the transcription of estrogen-responsive target genes in a dose-dependent manner (Bowers et al. 2000; Kuiper et al. 1997, 1998; Maggiolini et al. 2001). However, phytoestrogens bind the ER with much lower affinity compared with E _2_ (McCarty 2006; van der Woude et al. 2005). The affinity of quercetin for ER-α and ER-β was shown to be 105- to 106-fold lower than the affinity of E_2_ for ER-α and ER-β (van der Woude et al. 2005). Similarly, the affinity of daidzein for ER-α and ER-β was found to be approximately 20,000- and 500-fold lower than that of E_2_ (Lehmann et al. 2005)

Unlike E_2_, which binds both ER-α and ER-β with similar affinity, many phytoestrogens display a substantially higher affinity for ER-β. For example, the binding affinities of genistein and daidzein for ER-β were shown to be significantly higher than for ER-α (Kuiper et al. 1997, 1998; Lehmann et al. 2005). Moreover, phytoestrogens induce the transcription of estrogen-responsive target genes to much greater levels when bound to ER-β than when bound to ER-α. In MCF7 cells co-transfected with either ER-α or ER-β, genistein was shown to induce a 100-fold greater induction of gene expression when bound to ER-β than when bound to ER-α (Harris et al. 2005). This result is in agreement with other evidence that genistein preferentially binds ER-β and induces greater DNA-binding and transcriptional activity when bound to ER-β (An et al. 2001; Barkhem et al. 1998; Kostelac et al. 2003; Liu et al. 2003; Morito et al. 2001; Mueller et al. 2004; Routledge et al. 2000). Like genistein, resveratrol induced higher transcriptional activity in estrogen-responsive genes when bound to ER-β compared with ER-α (Bowers et al. 2000).

Despite, the significantly lower affinity of phytoestrogens for the ER compared with E_2_,some phytoestrogens reportedly induce ER-mediated gene transcription from both ER-α and ER-β to higher levels than E_2_ (Harris et al. 2005; van der Woude et al. 2005). The reported maximal inductions of gene transcription from ER-α by genistein and quercetin were 1.4- and 1.7-fold greater than those produced by E_2_ and 2.4- and 4.5-fold greater than those produced by E_2_ for ER-β (van der Woude et al. 2005). Similarly, the maximum induction of ER-mediated genes by quercetin for ER-α and ER-β were 1.7 and 4.5 times greater than those reached by E_2_ (van der Woude et al. 2005). In addition, the presence of endogenous estrogens has been shown to influence the effect of phytoestrogens on gene transcription. For example, both genistein and resveratrol were found to act synergistically with E_2_ to activate ER-α– and ER-β–induced gene transcription in MCF7 breast cancer cells (Gehm et al. 1997; Harris et al. 2005).

Because phytoestrogens have significantly different affinities for ER-α and ER-β, the net effect of exposure to a particular phytoestrogen may depend on the distinctive patterns of ER-α and ER-β expression in different cell types (McDonnell 2004). The differential affinities of phytoestrogens for ER-α and ER-β suggest that physiologic concentrations of phytoestrogens may be enough to activate ER-β but not ER-α, implying that rather than acting via the classical ER-α pathway, phytoestrogens may activate ER-β and induce its antiproliferative effects (Figure 2; Appendix 1) (McCarty 2006). Moreover, the presence of type II sites in the breast and uterus adds another dimension of complexity to phytoestrogen action. Type II sites, low-affinity nuclear binding sites for E_2_ in the breast and uterus, are suggested to be involved in regulating the growth and proliferation of both normal and malignant cells (Markaverich et al. 2001; Shoulars et al. 2002, 2005). Type II sites have not been fully characterized, although histone H4 binds type II ligands and is thought to be the type II site (Markaverich et al. 2001; Shoulars et al. 2002, 2005). Phytoestrogens, specifically flavonoids such as quercetin, bind type II sites with high affinity and antagonize growth in a number of cell types, suggesting another mechanism by which phytoestrogens may modulate cell proliferation (Griffiths and Smith 1972; Markaverich et al. 2001; Shoulars et al. 2002, 2005). Although many studies have been performed, a more detailed understanding of how phytoestrogens interact with the estrogen receptor is critical to fully evaluate their toxicologic and pharmacologic properties.

## Phytoestrogens: Cellular Growth and Proliferation

Evidence that phytoestrogens can activate the estrogen receptor and may mimic endogenous estrogens has raised concerns regarding their effects on cell growth and proliferation. If phytoestrogens, like estrogens, promote cell growth, they may stimulate the expansion of pre-existing tumors (Figure 2; Appendix 1). However, the distinctive activities of the ER isoforms as well as the differential affinities of low concentrations of phytoestrogens for ER-β over ER-α suggest that the net effect of phytoestrogen exposure on cell growth may be quite different from those of estrogen on the classic ER system (McCarty 2006).

Many phytoestrogens appear to have a biphasic effect on cell proliferation, stimulating growth at low concentrations and suppressing growth at high concentrations. At low concentrations, resveratrol and quercetin dose dependently promoted growth in ER-positive MCF7 cells but inhibited proliferation and induced cell death at high concentrations (Ashby et al. 1999; Bowers et al. 2000; Gehm et al. 1997; Lu and Serrero 1999; Maggiolini et al. 2001; Schmitt et al. 2002; van der Woude et al. 2005). Similarly, genistein was shown to increase growth in estrogen-sensitive cells at low concentrations but decreased cell growth, suppressed DNA synthesis, and induced cell death at high concentrations (Maggiolini et al. 2001; Seo et al. 2006). A recent study revealed that the proliferation observed in daidzeintreated MCF7 cells was blocked by the pure antiestrogen ICI 182,780, indicating that the stimulatory effect exerted by daidzein was ER-mediated (Ju et al. 2006b). While phytoestrogens promote cell growth in ER-positive cells, evidence suggests that ER-negative cells may have different responses to phytoestrogen exposure. In the ER-negative breast cancer cell line MDA-MB-468, resveratrol inhibited cell proliferation at all concentrations lower than 10 nM (Ashby et al. 1999; Bowers et al. 2000; Gehm et al. 1997; Lu and Serrero 1999; Schmitt et al. 2002). Similarly, low concentrations of quercetin and genistein reduced proliferation or had no stimulatory effect on ER-negative MDA-MB-231, HCC-38, and HeLa cells (Balabhadrapathruni et al. 2000; van der Woude et al. 2005).

The effects of phytoestrogens on the growth and proliferation of tumor cells have also been evaluated *in vivo*, using animal models of breast cancer and breast cancer cell xenografts. Genistein and soy protein stimulated the growth of MCF7 breast cancer cell xenografts implanted in mice (Allred et al. 2001; Hsieh et al. 1998). Similarly, genistein, in the presence of low levels of E_2_, acted in an additive manner to stimulate the growth of MCF7 tumors in mice (Ju et al. 2006a). However, other studies have generated conflicting results. Genistein inhibited the growth of both ER-positive and ER-negative breast cancer xenografts and induced apoptosis in tumor cells (Shao et al. 1998). Similarly, resveratrol reduced tumor growth and increased apoptosis in ER-α–negative/ER-β–positive MDA-MB-231 tumor xeno-grafts (Garvin et al. 2006).

The effects of phytoestrogens on cell growth and proliferation may be explained by their ability to alter the expression of a number of proteins that control cell cycle and induce cell cycle arrest and apoptosis. In MCF7 and MDA-MB-231 cells, resveratrol caused cells to accumulate in the S-phase and down-regulated *Bcl-2*, resulting in apoptosis (Pozo-Guisado et al. 2002, 2005). The effect of resveratrol on the cell cycle is suggested to be mediated by its opposing effects on cell cycle regulators. Resveratrol increased the expression and activity of positive G_1_/S and G_2_/M cell cycle regulators, while simultaneously increasing protein levels of p21, p53, and p27 (Pozo-Guisado et al. 2002). Similarly, both genistein and quercetin caused G_2_/M arrest and apoptosis in MDA-MB-231 cells (Balabhadrapathruni et al. 2000). Increased cyclin B1 protein levels were observed in MDA-MB-231 cells following exposure to low doses of genistein, but MDA-MB-231 cells exposed to high concentrations of genistein displayed decreased levels of cyclin B1 and phosphorylated Cdc2 (Balabhadrapathruni et al. 2000). Further, daidzein was shown to alter cell cycle distribution and induce apoptosis in HeLa cells (Guo et al. 2004).

To resolve the dilemma regarding the potential beneficial or harmful effects of phytoestrogens in breast cancer development, numerous studies have attempted to characterize the estrogenic and growth-stimulatory actions of phytoestrogens. Most of these studies have been carried out in transformed breast cancer cell lines. To further our understanding of the proliferative effects of phytoestrogens, studies need to be performed in both nontumorigenic and tumorigenic breast cells with varying ER status and in environments with varying estrogen concentrations.

## Phytoestrogens and Tumor Development

Various phytoestrogens have been evaluated for their ability to prevent chemically induced mammary carcinogenesis. Resveratrol blocked the formation of preneoplastic lesions, suppressed mammary carcinogenesis, reduced tumor incidence, and increased tumor latency in Sprague-Dawley rats treated with dimethylbenz[*a*]anthracene (DMBA) (Whitsett et al. 2006). Both resveratrol and quercetin have been shown to inhibit *N*-methyl-*N*-nitrosourea (NMU) and DMBA-induced mammary carcinogenesis in rats (Banerjee et al. 2002; Bhat et al. 2001; Verma et al. 1988). Resveratrol decreased NMU- and DMBA-induced tumor incidence and multiplicity by 50% in Sprague-Dawley rats (Banerjee et al. 2002; Bhat et al. 2001). Daidzein inhibited DMBA-induced mammary tumors in rats and significantly increased tumor latency in mouse mammary tumor virus-neu mice (Constantinou et al. 2001; Jin and MacDonald 2002). Similarly, several studies have demonstrated that rats exposed to genistein early in life have a decreased incidence of DMBA-induced mammary tumors in adulthood (Fritz et al. 1998; Hilakivi-Clarke et al. 1999; Lamartiniere et al. 1998; Murrill et al. 1996). However, others have reported that genistein increased tumor cross-sectional area, increased tumor multiplicity, elevated the percentage of proliferative cells in tumors and increased the weight of estrogen-dependent mammary adenocarcinomas in rat models of mammary cancer (Allred et al. 2004; Kijkuokool et al. 2006).

In addition, phytoestrogen exposure has been shown to alter breast development (Figure 2; Appendix 1). Resveratrol-exposed female rats displayed more differentiated lobular structures and decreased proliferation in the mammary terminal ductal structures, making them less vulnerable to damage by carcinogens (Whitsett et al. 2006). Exposure to genistein during breast development altered breast morphology and resulted in decreased terminal ductal formation (Hilakivi-Clarke et al. 1999). However, the relationship between phytoestrogens and tumor development does not always appear to be protective and may rely on age at exposure and the hormonal environment. Quercetin potentiated the severity of E_2_-induced kidney tumorigenesis in male Syrian hamsters, and prepubescent rats treated with resveratrol showed accelerated NMU-induced mammary carcinogenesis and elevated tumor incidence and multiplicity (Sato et al. 2003; Zhu and Liehr 1994).

Taken together, these studies indicate that certain phytoestrogens might reduce the risk of chemically induced mammary cancers in animal models, particularly if exposure is early in life. However, very few studies have focused on the effect of phytoestrogens on estrogen-induced breast cancers, which would be the most relevant model for gaining insight into the relationship between phytoestrogens and human mammary carcinogenesis.

## Phytoestrogens and Signaling Pathways

The influence of ERs on the transcription of estrogen-sensitive genes is not limited to ERE binding. An increasing body of evidence suggests that both ER-α and ER-β participate in some of the signaling cascades responsible for controlling gene expression, cell cycle, cell proliferation and apoptosis (Levin 1999; Marquez and Pietras 2001; Pietras et al. 2005a, 2005b). Several phytoestrogens have been shown to modulate the activity of ER-associated signaling cascades and transcription factors. In human breast cells, resveratrol inhibited ER-α–associated PI3K activity, thereby exerting an inhibitory effect on cell proliferation and survival (Pozo-Guisado et al. 2005). Genistein and daidzein activated Akt in the ER-α–positive T47D breast cancer cell line, whereas resveratrol inhibited Akt phosphorylation (Brownson et al. 2002). In the ER-α–negative MDA-MB-231 breast cancer cell line, resveratrol and daidzein activated Akt but genistein did not (Brownson et al. 2002). Resveratrol has been shown to modulate nuclear factor kappa B (NFκB) and AP-1 activation in various cancer cell lines, leading to the speculation that NFκB and AP-1 are potential targets of resveratrol (Banerjee et al. 2002; Kundu and Surh 2004). In MCF7 cells and chemically induced rat mammary tumors, resveratrol inhibited the DNA-binding activity of NFκB (Banerjee et al. 2002). Resveratrol inhibited extracellular signal–regulated kinase (ERK) and p38 mitogen-activated protein kinase (MAPK) activation in mouse skin cells, suggesting that resveratrol may inhibit the activation of NFκB and AP-1 at the level of their upstream kinases, ERK and p38 MAPK (Yu et al. 2001). Similarly, genistein blocked the NFκB signaling pathway via an Akt-dependent mechanism in both MDA-MB-231 breast cancer cells and PC3 prostate cancer cells (Gong et al. 2003; Li and Sarkar 2002). Genistein and daidzein suppressed NFκB activation in TNFα-stimulated mouse fibroblasts and in ER-negative breast cancer cells by a mechanism that involved abrogation of MEK1 and ERK activity (Vanden Berghe et al. 2006). Treatment of mouse fibroblasts with the antiestrogen ICI 182780 failed to reverse the effects of daidzein and genistein on NFκB-dependent gene expression, indicating that suppression of NFκB is independent of the estrogenic activity of phytoestrogens (Vanden Berghe et al. 2006).

Recently, microarray technologies have been exploited in order to clarify the estrogenic effects of phytoestrogen exposure on gene expression and signaling pathways (Chen et al. 2003; Ise et al. 2005; Naciff et al. 2002). One such study compared the effects of genistein and E_2_ exposure on the reproductive tissues of the developing rat fetus, specifically the ovaries and uterus (Naciff et al. 2002). Expression patterns of genes whose products were involved in cell growth, differentiation, stress response and apoptosis were modulated by both genistein and E_2_. However, genistein exposure altered the expression patterns of a number of genes in a manner distinct from E_2_ (Naciff et al. 2002). Genistein increased the expression of MAPK and topisomerase II, whereas the expression of phospholipase A_2_ was down-regulated. In contrast, the expression of these genes was not affected by E_2_ exposure (Naciff et al. 2002). Another study, in which MCF7 breast cancer cells were treated with genistein, showed down-regulation of genes whose products are associated with cell growth, DNA replication, and growth factor response (Chen et al. 2003). In a separate study, gene expression profiles in MCF7 breast cancer cells were evaluated after exposure to either phytoestrogens or E_2_ (Iseet al. 2005). The authors reported similar but distinct expression patterns for each of the phytoestrogens tested and analyses revealed that the phytoestrogen exposure induced expression profiles with differing degrees of similarity to E_2_ (Ise et al. 2005).

## Phytoestrogens and Estrogen-Metabolizing Enzymes

Once ingested, phytoestrogens interact with many of the same enzymes as endogenous estrogens and have been shown to interfere with the process of estrogen metabolism (Moon et al. 2006). Several phytoestrogens are known to modify the CYP450 enzyme system by either inducing or suppressing the transcription of CYP450 enzymes or by inhibiting or enhancing enzyme activity (Figure 2; Appendix 1) (Moon et al. 2006). The expression of two main estrogen-metabolizing enzymes, Cyp1A1 and Cyp1B1, is under the control of the aryl hydrocarbon receptor (AhR), a ligand-activated transcription factor, which binds a specific DNA sequence designated xenobiotic response element (XRE) in the promoter regions of its target genes (Nebert et al. 2000).

Resveratrol, genistein and quercetin have been shown to decrease both xenobiotic-induced transcription and activity of Cyp1A1 and Cyp1B1 in numerous cell types (Berge et al. 2004; Chan et al. 2003; Chang et al. 2001; Chun et al. 1999; Ciolino and Yeh 1999; Han et al. 2006; Lee and Safe 2001; Ramadass et al. 2003; Roberts et al. 2004; Shertzer et al. 1999). Resveratrol is an AhR antagonist and is suggested to exert its inhibitory effects on *Cyp1A1* and *Cyp1B1* expression either by suppressing AhR DNA-binding activity or by preventing the interaction between AhR and the transcriptional complex, thereby blocking induction of AhR-mediated genes (Casper et al. 1999; Chen et al. 2004). Similarly, genistein and quercetin are suspected to decrease xenobiotic-induced *Cyp1A1* and *Cyp1B1* mRNA expression by interfering with activation of the XRE by AhR (Chan et al. 2003; Ramadass et al. 2003). In contrast, quercetin has been shown to both increase and decrease Cyp1A1 enzyme activity (Ciolino et al. 1999; Ramadass et al. 2003).

Not only do phytoestrogens interact with the Cyp450 enzyme system, quercetin inhibits the *O*-methylation of endogenous estrogens by catechol-*O*-methyltransferase (COMT) by a combination of three mechanisms—direct competition for COMT, noncompetitive inhibition via an increase in *S*-adenosyl-L-homocysteine (SAH) concentrations and by reducing the availability of the methyl donor *S*-adenosyl methionine (SAM) (Zhu 2002). Various studies have established that quercetin is an excellent substrate for COMT, having a metabolic rate up to 30 times higher than catechol estrogens (Zhu 2002). In Syrian hamsters treated with E_2_, quercetin increased the concentrations of 2-hydroxyestradiol (2-OHE_2_) and 4-hydroxyestradiol (4-OHE_2_) in kidney and decreased urinary excretion of 2-methoxyestradiol (2-MeOHE_2_) and 4-methoxyestradiol (4-MeOHE_2_) (Zhu and Liehr 1996).

The evidence that phytoestrogens alter estrogen-metabolizing enzymes is not limited to *in vitro* data. Data from nonhuman primate studies suggest that exposure to phytoestrogens alters the pathways of estrogen metabolism by Cyp1A1 and Cyp1B1 *in vivo* such that it is shifted toward the production of fewer genotoxic metabolites (Lu et al. 2000b; Wood et al. 2006). However, inhibition of COMT by phytoestrogens would not only lead to elevated tissue levels of the procarcinogenic estrogen metabolite 4-OHE_2_, but also to decreased levels of the anti-carcinogenic estrogen metabolite 2-MeOHE_2_ (Badawi et al. 2001; Zhu 2002). Thus, it is

## Conclusion

Epidemiologic evidence has been inconclusive regarding the effects of phytoestrogen consumption on breast cancer risk. Nevertheless, extensive research has been performed to provide a detailed description of the possible mechanisms of action of phytoestrogens. Taken together, the research on phytoestrogens, particularly studies relevant to genistein, daidzein, resveratrol, and quercetin, suggests that these compounds do not act by a single mechanism to achieve their effects. Instead, these plant substances exert their effects by way of various mechanisms, including effects on estrogen-metabolizing enzymes, cell cycle, cell differentiation, proliferation, apoptosis, the inflammatory response and various cell signaling pathways.

While there is some evidence supporting a chemoprotective role for phytoestrogens in breast cancer, there is also evidence suggesting the possible adverse effects of phytoestrogen consumption. More research is needed in order to fully evaluate the activities of phytoestrogens and the biological relevance of experimental findings. Future studies may focus elucidating the mechanisms underlying phytoestrogen action and to characterizing the actions of phytoestrogens in different hormonal environments.

## Appendix 1. Overview of possible mechanisms of phytoestrogen action

### Putative anticancer actions

- Inhibition of HSD and Cyp19 to decrease endogenous estrogen levels
- Modulation of Cyp450s to reduce ratio of genotoxic estrogens to total estrogens
- Activation of ER-β to exert antiproliferative and prodifferentiative effects
- Reduction of mammary sensitivity to carcinogens by altering breast development/morphology

### Putative procancer actions

- Stimulation of growth and proliferation of breast epithelial cells via ER-α activation
- Stimulation of growth and proliferation of existing tumors via ER-α activation important for future studies to further elucidate the abilities of phytoestrogens to alter the metabolism of endogenous estrogens, as these processes are critical to understanding the relationship between phytoestrogens and breast cancer development.
